# Effects of Aqueous Extract of *Lycopersicum esculentum* L. var. “Camone” Tomato on Blood Pressure, Behavior and Brain Susceptibility to Oxidative Stress in Spontaneously Hypertensive Rats

**DOI:** 10.3390/pathophysiology28010012

**Published:** 2021-03-18

**Authors:** Maria Frosini, Paola Marcolongo, Alessandra Gamberucci, Gabriella Tamasi, Alessio Pardini, Roberta Giunti, Paolo Fiorenzani, Anna Maria Aloisi, Claudio Rossi, Federica Pessina

**Affiliations:** 1 Department of Life Sciences, University of Siena, 53100 Siena, Italy; maria.frosini@unisi.it; 2 Department of Molecular and Developmental Medicine, University of Siena, 53100 Siena, Italy; alessandra.gamberucci@unisi.it (A.G.); roberta.giunti@unisi.it (R.G.); 3 Department of Biotechnology, Chemistry and Pharmacy, University of Siena, 53100 Siena, Italy; gabriella.tamasi@unisi.it (G.T.); alessio.pardini@unisi.it (A.P.); claudio.rossi@unisi.it (C.R.); 4 Department of Medicine, Surgery and Neurosciences, University of Siena, 53100 Siena, Italy; paolo.fiorenzani@unisi.it (P.F.); annamaria.aloisi@unisi.it (A.M.A.)

**Keywords:** Camone tomato, tomatine, open field, behavior, brain, spontaneously hypertensive rats (SHRs), blood pressure

## Abstract

Behavioral disorders affect millions of people worldwide. Hypertension contributes to both the development and progression of brain damage and cognitive dysfunction and could represent the most powerful modifiable risk factor for cerebral vessel dysfunction and consequent behavioral impairment. Tomato contains antioxidants and bioactive molecules that might play an important role in the prevention of cardiovascular and brain diseases. The effects of the combined gel and serum from *Lycopersicum esculentum* L. var. “Camone” tomatoes and those of purified tomato glycoalkaloids (tomatine) and an antihypertensive drug (captopril) were investigated in male spontaneously hypertensive rats (SHRs) and compared with normotensive Wistar Kyoto (WKY) rats. Body weight, systolic blood pressure, behavioral parameters, as well as brain susceptibility to oxidative stress and brain cytokine contents, were assessed. Treating hypertensive rats with tomato gel/serum or captopril for four weeks caused a significant reduction in blood pressure, decreased locomotor activity and increased grooming behavior; the last two parameters were also significantly affected by tomatine treatment. Brain slices obtained from hypertensive rats treated with tomato gel/serum were more resistant to oxidative stress and contained lower levels of inflammatory cytokines than vehicle-treated ones. In contrast, tomatine treatment had no effect. In conclusion, the tomato-derived gel/serum can be considered a dietary supplement able to drive in vivo blood pressure towards healthier values and also control some central effects such as behavior and brain oxidative stress.

## 1. Introduction

Over the past two decades, there has been increasing interest in the potential health benefits associated with the intake of nutraceuticals and functional food [1,2]. In previous papers, it was demonstrated that the locular gel and serum from the Camone tomato variety, which often represents waste for tomato industries, contains high amounts of glycoalkaloids (α-tomatine and dehydrotomatine) and polyphenols, of which chlorogenic acid is the most abundant [3,4]. Many reports have highlighted the benefits of these bioactive compounds, either isolated or in combined extracts, depending on dose and conditions of use. For example, tomato glycoalkaloids possess cardioprotective, anticholesterolemic, antidiabetic, hepatoprotective and neuroprotective properties [5]. These benefits mostly depend on their well-known antioxidant [6] and anti-inflammatory activities, suggesting that tomato-based foods might decrease the risk of many chronic diseases.

Several studies have reported that the intake of foods containing vitamins, flavonoids and phenolic compounds is associated with a lower prevalence of cardiovascular and brain diseases [7,8], in which inflammation and reactive oxygen species (ROS) formation are key features of their heterogeneous pathophysiologic mechanisms. Primary arterial hypertension is a frequent health problem worldwide and is increasing in prevalence [9]. With time, high blood pressure, if not lowered with appropriate therapy and a correct lifestyle, impairs cognitive function, increases left ventricular mass and causes end-stage renal disease and arteriosclerosis [10]. Hypertension also increases the susceptibility of the brain to ischemic injury, as it alters the structure of cerebral blood vessels and disrupts intricate regulatory mechanisms that ensure an adequate blood supply to the brain [11]. Thus, a dietary approach could be an ideal means to support drug therapy in hypertension, particularly in patients with concomitant cardiovascular risk factors.

While high blood pressure is believed to be most common in adults, hypertension among young people is frequent [12] and children with sustained primary hypertension often develop attention deficit hyperactivity disorder (ADHD) [13]. Unfortunately, ADHD medication is associated with several side-effects and long-term consequences. Hence, there is considerable interest in the development of alternative treatment options. Among these, the possibility that a nutritional approach might positively impact ADHD by affecting the complex interplay between the microbiota, gut and brain has been proposed [14].

Spontaneously hypertensive rats (SHRs) are a commonly used model of both human essential hypertension (as their blood pressure increases spontaneously at ten weeks of age) and ADHD (because of their elevated locomotor activity) [15,16]. However, it is not clear whether the pathways involved in the development of hypertension overlap with those involved in the modulation of increased locomotor activity.

Therefore, the aim of this study was to investigate whether four weeks of treatment with the combined gel and serum (Gs, obtained from green tomatoes of the Camone variety), in addition to counteracting the spontaneous increase in blood pressure in SHRs, as previously demonstrated [4], could affect locomotor activity and other spontaneous behaviors; moreover, brain susceptibility to oxidative stress and brain cytokine contents were assessed in the same experimental condition. Pure tomatine (of which Camone tomato is particularly rich) was also tested.

## 2. Materials and Methods

### 2.1. Materials

*Lycopersicum esculentum* L. var. “Camone” was chosen on the basis of a previous chemical characterization [4]. Tomatoes were obtained from the same farm (Pachino, Sicily, Italy) and purchased in the same month (May). Tomato locular gel and the serum were gently separated, avoiding seeds and pulp impurities, as previously described [4]. The gel was then lyophilized (5Pascal LIO-5P freeze-dryer, (Millrock Thecnology, Inc., NY, USA); −51 ± 2 °C, 1.3 ± 0.3 mbar, 5 days), finely powdered in a mortar and stored in the dark at −20 ± 1 °C until use. The serum was collected in polyethylene containers and stored under the same conditions. For in vivo studies, aliquots of lyophilized gel from each stock of tomatoes were reconstituted at 1:30 weight/volume with serum (gel/serum, Gs). The serum and locular gel samples of Camone tomato were extracted in triplicate by optimized solid–liquid ultrasound-assisted protocols [3] and chemically characterized as previously reported [3,4].

### 2.2. Animal Housing and Treatments

This study complied with European legislation on the use and care of laboratory animals (EU Directive 2010/63) and was approved by the Animal Ethics Committee of the University of Siena and the Italian Health Ministry (no. 185/2015PR). All efforts were made to minimize the number of animals used and their suffering.

Nine-week-old male spontaneously hypertensive rats (SHRs) and Wistar Kyoto (WKY) rats were purchased from Charles River (Charles River S.p.A, Lecco, Italy.). Animals were caged in groups of four and allowed to settle down for a week in conditions of controlled temperature, alternate 12 h light–dark cycle and ad libitum availability of food and water. A standard pellet diet was used (53.5% carbohydrates, 18.5% protein, 3.0% fat; 6% crude fiber; 7% crude ash; 12% humidity; 4RF21 Mucedola, Milan, Italy).

**Figure 1:**
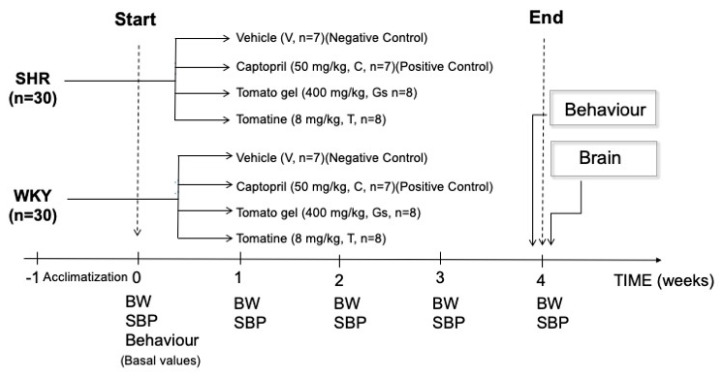
After a one-week housing period (acclimatization period) both Wistar Kyoto (WKY) and spontaneously hypertensive rats (SHRs) were randomly assigned and treated for four weeks, during which body weight (BW) and systolic blood pressure (SBP) were measured on a weekly basis. Basal SBP and basal weight were obtained just before starting any treatments, while behavioral parameters were assessed on the day before the treatments (basal behavior) and on the day before the end of the treatments (sacrifice day). After the animals were sacrificed, the brains were collected for ex vivo experimentation on brain slices.

At ten weeks of age, when hypertension starts to develop [16], SHRs were randomly divided into four groups and treated daily by oral gavage (volume 3 mL) for four weeks with Gs, tomatine or captopril, with the angiotensin-converting enzyme (ACE) inhibitor being adopted as positive control. Vehicle treatment (physiological solution) was used as negative control. Normotensive Wistar Kyoto rats (WKY) were used as a matched-age control strain (Figure 1).

Age-matched WKY animals were used as paired controls (V; C; Gs; T).

The treatment with tomato gel/serum (Gs) consisted of tomato lyophilized gel (100 mg) reconstituted in serum (3 mL) resulting in 12.4 g/kg (n = 8). This preparation allowed us to gavage the rats with the following major components: α-tomatine—396 ± 7, 54 ± 2, 78 ± 2 and 25 ± 1; chlorogenic acid—1276 ± 37, 1470 ± 58, 832 ± 15 and 431 ± 10; caffeic acid—48 ± 0.5, 116 ± 6, 72 ± 3 and 78 ± 1 μg/kg body weight of rat (in Week 1, Week 2, Week 3 and Week 4, respectively).

The purified tomatine (natural mixture of α-tomatine and dehydrotomatine) was purchased from TCI Chemicals Europe (Zwijndrecht, Belgium) and its composition was analyzed, corresponding to 87 ± 2% and 13 ± 1% of the two glycoalkaloids, respectively [3].

Every week, the rats were briefly removed from their cages and weighed. At the end of the four weeks, they were fully anesthetized by intraperitoneal injection of 15 mg/kg of Zoletil 100^®^ (Virbac Srl, Milano, Italy) and 4 mg/kg of Xylor^®^ (Bio 98, San Lazzaro, Italy) and killed by decapitation.

#### 2.2.1. Blood Pressure Measurement

Systolic blood pressure (SBP) was measured once a week with the noninvasive “tail-cuff” method [4]. At the time of measurement, three consecutive SBP readings were taken in the morning between 09:00 a.m. and 01:00 p.m. after warming the body of the rat at 37 °C for 5 min.

Basal SBP values were obtained previously during the one-week acclimatization period by measuring the rats each day to habituate them to the procedure.

#### 2.2.2. Open Field Test

The open field test was carried out in the open field apparatus, a square transparent Plexiglas cage (50 × 50 cm) with 30 cm high walls situated on a platform 80 cm above the floor. The rat was kept in the open field for 15 min between 08:30 a.m. and 11:00 a.m. and its locomotion and spontaneous behaviors (see below) were observed, recorded and analyzed with Observer behavioral software (Noldus Information Technology, Amsterdam, The Netherlands) by a researcher blind to the treatment [17].

All rats were tested the day before the beginning of the four weeks of treatment (basal behavior) and one day before the end of the experiment—i.e., one day prior to SBP measurement—in order to avoid the effect of the open field test on SBP levels.

Four separate behavioral responses were assessed in the open field: time duration of locomotion, grooming and crouch and frequency of rearing. Rearing behavior, consisting of the animal standing on both hind paws in a vertical upright position, sniffing, raising its head and moving its vibrissae, is considered a manifestation of searching activity. Behavioral events such as washing, licking the body and scratching were classified as grooming. The state in which the rat leaned on the floor with all four limbs without moving within the squares and without raising its head was considered as a crouch.

Behaviors were recorded by direct visual observation of three 5-min periods (total of 15 min per animal) and these three periods were averaged together.

#### 2.2.3. Brain Cortical Slices

Brain cortical slices were prepared according to standard experimental protocols [18]. Briefly, the brain was quickly washed in artificial cerebrospinal fluid (ACSF) (composition in mM: 120 NaCl; 2 KCl; 1 CaCl2; 1 MgSO4; 25 HEPES; 1 KH2PO4; 10 glucose, pH 7.4 and previously bubbled with a 95% O2–5% CO2 gas mixture for 20–30 min) and then the cortex was dissected and cut into 400 µm-thick slices with a manual chopper (Stoelting Co., Wood Dale, IL, USA). Slices were transferred into sterile 24-well culture plates containing 0.5 mL ACSF/well previously filtered by passage through a 0.2 μm sterile filter and left at room temperature (25 °C) for 60 min to recover from slicing trauma (equilibration phase). During this period, the medium was removed and replaced with fresh oxygenated filtered ACSF every 15 min (equilibration phase). After the equilibration phase, oxidative stress was induced by treating the tissue with 5 mM H2O2 for 1 h. Hydrogen peroxide was freshly prepared from 30% stock solution prior to each experiment.

At the end of the treatment, the colorimetric MTT method was used to assess tissue viability. In particular, slices were washed with ACSF, incubated with 0.5 mg/mL of MTT (300 μL in the same 24-well plate), followed by incubation at 37 °C for 45 min. Then, slices were gently transferred into a 96-well plate, treated with 200 μL of DMSO and incubated for 30 min at 37 °C on a shaking plate. After incubation, 100 μL of supernatant was sampled and the formazan product was estimated at 560 and 630 nm (OD560–OD630). Slice viability was expressed as a percentage of untreated slices (controls).

#### 2.2.4. Brain IL-6, IL-1β and TNF-α Content

IL-6 (Millipore^®^, Milano, Italy), IL-1β (Thermo Fisher Scientific, Rodano, MI, Italy) and TNF-α (Boster PicoKine^TM^ ELISA, Boster Biological technology, Pleasanton, CA, USA) were measured according to the manufacturers’ instructions in brain homogenates. In particular, half brain hemispheres were quickly frozen in liquid nitrogen and stored at −80 °C until analysis. The day of the assay, the brains were thawed, homogenized in cold phosphate buffer 0.1 M pH 7.4 (tissue weight: buffer volume = 1:10) and centrifuged at 10,000 g × 10 min at 4 °C. Afterward the supernatant was collected and used for the ELISA assay, whose sensitivity and assay range were as follows: TNF-α sensitivity < 1 pg/mL, assay range: 7.8–500 pg/mL; IL-6: sensitivity 5.3 pg/mL, assay range 18.8–1200 pg/mL; IL-1ß sensitivity < 12 pg/mL, assay range: 25.6–2500 pg/mL. Values were normalized to the protein content of the sample.

### 2.3. Statistical Analyses

Results are reported as mean ± SEM. All data were compared for statistical significance by ANOVA followed by Bonferroni post-hoc test, as appropriate (GraphPad Prism version 5.04, GraphPad Software Inc., San Diego, CA). In all comparisons, the level of statistical significance (*P*) was set at 0.05.

## 3. Results

### 3.1. Effects of Tomato Gel/Serum, Tomatine and Captopril on Body Weight and Systolic Blood Pressure of SHRs and WKY Rats

No changes in skin, fur, eyes, mucous membranes or salivation were observed in the tomato Gs or other treated groups, nor were there tremors or death, suggesting an apparently safe profile of the treatments. The tomato Gs, tomatine and captopril treatments did not significantly affect growth rate, as weight gain of these groups was comparable to that of rats treated with vehicle (final weight gain about 40 g, see Table 1).

**Figure 2:**
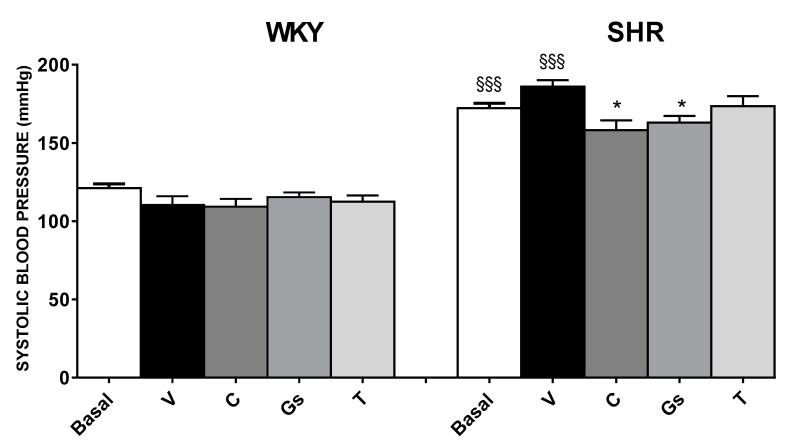
SHRs (right) and WKY (left) rats were treated for four weeks with vehicle (V), captopril (C) (50 mg/kg), tomatine (T) (8 mg/kg) or tomato gel/serum (Gs) (400 mg/kg). Basal: values recorded at the beginning of the four weeks of treatment. Data are reported as mean ± SEM. §§§ *p* < 0.001 vs. same treatment in WKY; * *p* < 0.05 vs. SHR V (ANOVA followed by Bonferroni).

SBP was significantly higher in SHRs when compared to WKY rats at the end of four weeks of observations: 186 ± 4 vs. 110 ± 5 mmHg (*p* < 0.001) [4]. Captopril and tomato Gs significantly decreased SBP in SHRs (*p* < 0.05), while no significant effect was found for tomatine. Moreover, none of the treatments had any significant effects on SBP in WKY rats (Figure 2).

### 3.2. Open Field Behaviors

**Figure 3:**
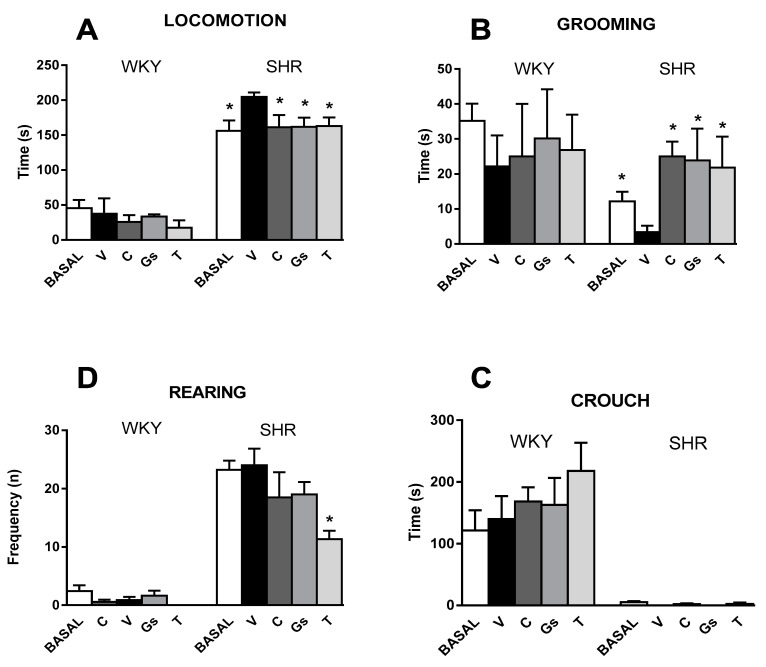
Time duration (s) of locomotion (**A**), grooming (**B**) and crouch (**C**) and frequency of rearing (**D**). SHRs (S) and WKY (W) rats were treated for four weeks with vehicle (V), captopril (C) (50 mg/kg), tomatine (T) (8 mg/kg) or tomato gel/serum (Gs) (400 mg/kg). Different behaviors were assessed at day 0 (before the treatment, BASAL) and at the end of any treatment. Values represent mean ± SEM; * *p* < 0.05 vs. SHR V; no significant differences were observed in the WKY groups (ANOVA followed by Bonferroni).

Before treatment, locomotion, grooming, rearing and crouch values of SHRs were generally different from those observed in WKY rats. In particular, locomotion was of longer duration (SHR—156 ± 10 vs. WKY—44.5 ± 7.9 s, *p* < 0.001, Figure 3A) and rearing frequency was higher (SHR—23.2 ± 3.5 vs. WKY—2.44 ± 0.54 s, *p* < 0.001, Figure 3C). Grooming lasted less over time (SHR—12.1 ± 2.1 vs. WKY—35.2 ± 9.6 s, *p* < 0.05, Figure 3B), while crouch was nearly nil (SHR—5.1 ± 1.2 vs. WKY—121 ± 28 s, *p* < 0.001, Figure 3D). The different treatments used in this study did not affect WKY behaviors over the four weeks. In contrast, changes were noticed in SHRs receiving captopril, tomato Gs and tomatine. They mainly consisted of a decrease in locomotor activity (approximately −16.0% for captopril, Gs and tomatine, *p* < 0.05 vs. SHR receiving vehicle) (Figure 3A) and a recovery of grooming duration (Figure 3B). The latter was more than halved in vehicle-treated SHRs after four weeks (basal SHR—7.9±1.8 vs. vehicle SHR—3.3±1.8 s, *p* < 0.01), while it regained values comparable to those observed in the normotensive strain for captopril, tomatine and tomato Gs treatments. Instead, no significant effect of any treatment was observed for rearing and crouch in either SHRs or WKY rats, with the exception of tomatine, which significantly lowered rearing behavior in SHRs (Figure 3C,D).

### 3.3. Oxidative Damage and Inflammatory Cytokine Contents of Brain Slices

**Figure 4:**
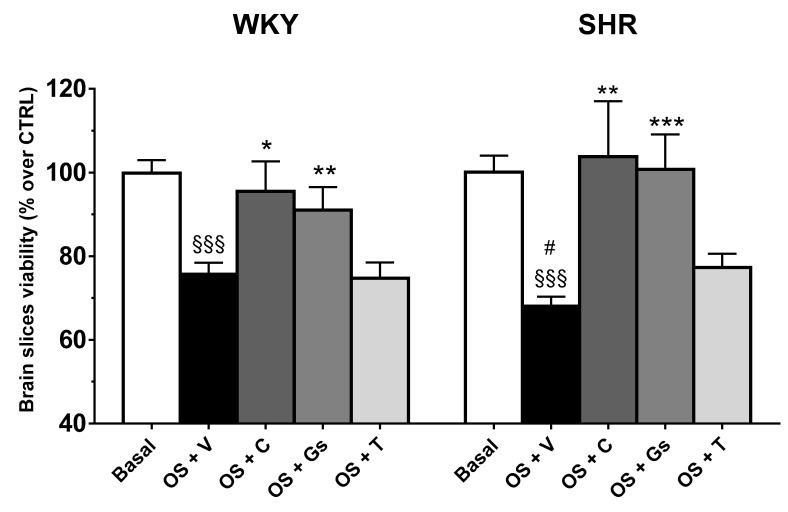
SHRs and WKY rats were treated for four weeks with tomato aqueous fraction (Gs, 400 mg/kg), tomatine (T, 8 mg/kg), captopril (C, 50 mg/kg) or vehicle (V). After the treatments, the brain was removed and cortical slices were subjected to oxidative stress (OS) (hydrogen peroxide 5 mM, 1 h). Tissue viability was assessed by MTT assay and expressed as percentage of untreated slices (Basal). Values are reported as mean ± SEM. ^§§§^ *p* < 0.001 vs. Basal. * *p* < 0.05, ** *p*  <  0.01, *** *p*  <  0.001 vs. OS+V. # *p* < 0.05 vs. OS+V in WKY (ANOVA followed by Bonferroni).

The brain is a major target of the deleterious effects of hypertension, and increasing evidence suggests that oxidative stress is involved in these harmful effects [11]. For this reason, we assessed whether brains of SHRs and WKY rats treated with tomato Gs, tomatine or captopril were (or not) less susceptible to oxidative stress-mediated injury. The results showed that hydrogen peroxide challenge significantly affected the viability of brain slices, with a more pronounced effect on those of SHRs (+8%, *p* < 0.05 vs. oxidative stress in WKY brain slices) (Figure 4).

Interestingly, treatment with tomato Gs and captopril, but not tomatine, exerted significant neuroprotection, as in both the WKY and SHR strains the viability of tissue after the injury was comparable to untreated slices.

**Figure 5:**
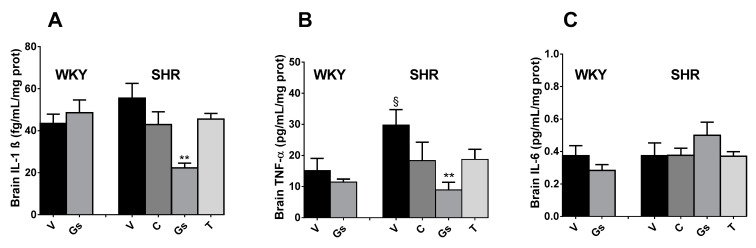
IL-1β panel (**A**), TNF-α panel (**B**) and IL-6 panel (**C**) contents measured in brains of SHRs or WKY rats treated for four weeks with tomato aqueous fraction and gel (Gs, 400 mg/kg), tomatine (T, 8 mg/kg) and captopril (C, 50 mg/kg). Animals treated with vehicle (V) represent the control. Values are reported as mean ± SEM. ** *p* < 0.01 vs. SHR V; § *p* < 0.05 vs. WKY V (ANOVA followed by Bonferroni).

Brain cytokine contents were also assessed since inflammation is involved in the pathogenesis of hypertension [19]. As reported in Figure 5, the brains of SHRs treated with vehicle contained higher amounts of TNF-α (+96.3%, *p* < 0.05 vs. WKY) and IL-1β (+27.9%, *p* < 0.05 vs. WKY). Tomato Gs, but not captopril or tomatine, restored basal values of the brain contents of these cytokines. Finally, IL-6 was mostly unaffected by all treatments in both WKY rats and SHRs.

## 4. Discussion

This study investigates the effect of long-lasting treatment with tomato Gs, tomatine and captopril in SHRs, at an age at which their SBP increases [16]. In addition to their antihypertensive effects demonstrated previously [4], the present study also points out their possible central effects. A strong interconnection between stress pathways in the brain and some elements of cardiovascular diseases, as well as increased blood–brain barrier permeability in SHR, have been reported [20,21]. Gs treatment, akin to that with captopril and to a lesser extent tomatine, significantly counteracted the blood pressure increase; moreover, for the first time, tomato Gs was demonstrated to reduce spontaneous locomotor hyperactivity and to normalize other behaviors in the open field. Interestingly, in brain slices, tomato Gs but not tomatine exerted neuroprotection against oxidative stress-mediated injury and reduced brain IL-1β and TNF-α contents. The experimental design involved a standard chow diet and a daily gavage treatment with tomato Gs, tomatine or captopril for four weeks. The rats were treated starting at ten weeks of age, when systolic blood pressure is already high and which increases with age. The rats were treated by oral gavage, ensuring the administration of accurate and reproducible amounts. Considering the chronic treatment to be performed (four weeks), the tomatine dose used was much lower than the toxic dose [4]. The study shows that prolonged treatment with Gs induced a significant decrease in SBP, similar to that caused by the traditional antihypertensive drug captopril, as compared to untreated SHRs. Tomatine decreased SBP, albeit not significantly. Previous studies have shown that Camone tomato fraction (the locular gel surrounding the seeds and the serum, normally discarded during industrial processing of tomato concentrate, tomato pulp and tomato sauce) is rich in bioactive compounds [3,4] such as the glycoalkaloid tomatine and chlorogenic and caffeic acids, important biologically active dietary polyphenols. Although it differs in chemical components from red tomatoes, the Camone variety has a higher antioxidant capacity [3,4].

Some authors have reported that the consumption of natural polyphenols results in an improvement of cognitive functions [22]. Studies in rodents demonstrated the variable effects of flavanols on animal behavior; oral epicatechin treatment was reported to significantly prevent a BP increase and to reduce behavioral hyperactivity in young SHRs [23]. However, to our knowledge, the effect of Gs and tomatine on behavior has never been investigated, while some authors reported positive effects of captopril on rat behaviors [24], in agreement with the results presented here. Moreover, lisinopril (an angiotensin-converting enzyme inhibitor) normalized the increase in SBP and partly reversed the alterations in anxiety-like behavior in SHRs [25]. The simultaneous reduction in hypertension and behavioral hyperactivity in young rats would be of interest, since high blood pressure occurs in children suffering from hyperactivity. An elevated activity in brain areas involved in both the increase in blood pressure and locomotor activity in SHRs with respect to normotensive rats has been reported [26].

Previous experiments performed in our laboratory, as well as studies by other researchers [4,23], have confirmed that the levels of locomotor and searching activities are higher in SHRs than in WKY rats. Interestingly, an attenuation of locomotor hyperactivity, as determined by a significant decrease in locomotion, was observed in SHRs receiving tomato Gs, tomatine and captopril with respect to SHRs receiving vehicle. Moreover, there was an increase in grooming with the same treatments, further demonstrating confident behavior and less hyperactivity in the animals and suggesting a correction of SHR behavioral abnormalities by tomato Gs and tomatine.

Rearing behavior was also attenuated by tomato Gs and tomatine, as well as captopril, albeit significantly only for tomatine, further suggesting less hyperactive behavior in the animals.

Increased locomotor activity has been used to model the positive symptoms of schizophrenia [27] because they are both associated with an increased dopaminergic activity in the mesolimbic pathway. The pharmacological modulation of locomotor hyperactivity in SHRs reinforces this suggestion; their hyperlocomotion is specifically reversed by antipsychotic drugs and potentiated by the psychostimulant amphetamine [28]. Thus, we cannot exclude that Gs might also be useful for the treatment of locomotor hyperactivity in schizophrenia. In adult SHRs, increased rearing behavior is diminished by the administration of antipsychotic drugs [28]. Peres et al. [29] recently reported that rearing behavior is highly correlated with a schizophrenia-like latent trait evaluated in adult WKY rats and SHRs. Interestingly, rearing behavior was only partially attenuated by tomato Gs but significantly by tomatine, further suggesting less hyperactive behavior in the animals and different mechanisms of action exerted by Gs and tomatine.

The simultaneous prevention of the SBP increase and reduced hyperactivity of SHRs observed in this study suggest the possibility of a common mechanism(s) underlying both pathologies. Indeed, Repova and coworkers [25] reported a lack of correlation between SBP and anxiety-like behavior in SHRs and lisinopril-treated SHRs, supporting the view that the behavioral modifications in these groups are not related to the hemodynamic changes. An association between behavioral modifications and neuro-humoral activation-hyperfunctional noradrenergic system in SHRs, similar to that in humans with essential hypertension, was suggested. In SHRs, captopril-induced changes in rat behaviors may be related to the sympatholytic action resulting from ACE inhibition [25]. Although the mechanisms by which tomato Gs affects behaviors and SBP need to be further investigated, we can speculate that Gs downregulates sympathetic activity, as already reported for some polyphenols [23,30], and thus hyperactive behavior. Chlorogenic acid, an important component of tomato Gs [4], has shown antioxidative and antihypertensive actions [31], as well as cognitive and neuroprotective effects [32]. More than one compound is present in tomato Gs, suggesting a possible synergic effect and more than one mechanism of action. Indeed, tomato Gs consumption seems to be a promising new therapeutic strategy to reduce the deleterious effects of essential hypertension both at the peripheral and central levels.

It is well known that in the hyperactive brain renin–angiotensin system (RAS), oxidative stress and neuroinflammation in brainstem cardiovascular centers and other brain regions increase sympathetic activity in hypertension [33]. The proinflammatory cytokines TNF-α, IL-1β and IL-6 act as neuromodulators in the paraventricular nucleus (PVN) of the hypothalamus by stimulating the production of cytotoxic ROS [34], which further propagate a sympatho-excitatory effect [35]. SHRs had higher IL-1β and TNF-α brain contents than the age-matched normotensive group, as previously described [36]. Instead, IL-6 was similar in SHRs and WKY rats, in contrast to what was previously observed at the PVN level [37]. When considering the present results, however, it cannot be ruled out that IL-6 is increased in a discrete area such as the PNV, but this was not detectable from measurement of cytokine contents in the whole brain. Interestingly, Gs, but not tomatine, restored the cytokine contents to basal values, while captopril was effective only towards IL-1ß. This observation indicates that the antihypertensive activity of Gs can be ascribed, at least in part, to its anti-inflammatory effect.

Chronic hypertension is accompanied by brain damage caused by a hypoxic/ischemic mechanism, in which oxidative stress plays a pivotal role [11,37]. High blood pressure values are also related to milder, subtle and chronic forms of brain damage, especially those controlling cognitive functions [38]. To this end, we assessed the susceptibility of rat brains treated with Gs, tomatine or captopril to oxidative stress-mediated injury. Interestingly, tissue viability was markedly improved in both SHRs and WKY rats treated with Gs and captopril, thus highlighting an interesting neuroprotective effect also in normotensive animals.

The most abundant polyphenols in tomato Gs are chlorogenic and caffeic acids as well as tomatine, whose neuroprotective properties have been described when used in the low μM range [39,40,41]. As the brain penetration of many polyphenols is still controversial, the possibility that tomato Gs components or their metabolites might cross the blood–brain barrier endothelium at physiologically relevant concentrations is intriguing. Very recently, however, it was reported not only that polyphenols might cross the BBB but that endothelial cells have an active role as they metabolize them into novel components, which have beneficial effects by modulating microglia-mediated inflammation [42]. Moreover, gut microbial metabolites of polyphenols, but not their parent compounds, may enter the blood, cross the BBB and provide neuroprotection, thus largely contributing to the polyphenol-mediated beneficial effects [43]. The present results strongly suggest that Gs (but not tomatine) can exert neuroprotective effects in both normotensive rats and SHRs—whether these effects depend on Gs components or their metabolites is a matter that needs to be further assessed.

Finally, captopril treatment also had a protective activity against oxidative stress-mediated injury in brain slices of both normotensive and hypertensive rats. Among the several potential mechanisms, the possibility that ACE inhibition leads to an increase in brain bradykinin which, in turn, reduces ROS formation [44] cannot be ruled out.

From the results of the present study, it is plausible that both the antihypertensive effect and the behavioral modifications shown by tomato Gs were due to more than one component or an additive effect of polyphenols and glycoalkaloids, which need to be administered for a long period of time, as often happens for healthy effects shown by natural nutrients.

Further studies are needed to elucidate the exact bioactive substance(s) and the exact site(s) of action of orally administered tomato Gs in preventing and treating hypertension and behavioral hyperactivity. The Gs used in the present study is a mixture of many compounds, among which alpha-tomatine, chlorogenic acid, caffeic acid are the most representative. Consequently, it is not easy to determine the mechanism of action underlying the Gs-mediated lowering of systolic blood pressure. In fact, natural compounds often affect multidimensional cellular networks at the same time, behaving as multitargeted compounds [45]. Another important issue is that the components of Gs might work in concert to achieve the observed biological effects or even to promote a synergistic interaction, although the possibility that some effects of active constituents are masked or reversed by other compounds present in the complex mixture cannot be ruled out. While the contribution of alpha-tomatine can be mainly ascribed to its anti-inflammatory and antioxidant activity [5], that of chlorogenic acid could depend on its ability to stimulate NO production through the endothelial-dependent pathway [46], inhibiting excessive production of ROS in the vasculature, leading to the attenuation of endothelial dysfunction, vascular hypertrophy and hypertension, as already observed in SHRs [47]. Indeed, both chlorogenic acid and tomatine can inhibit AChE and BChE [48,49], thus prolonging Ach-mediated vasodilatory effect in the artery and smooth muscle over time [50]. Moreover, chlorogenic acid, along with aqueous extract of tomatoes, also has ACE-inhibiting activity [46,51,52] or can downregulate the expression of ACE and renin [53]. In light of these considerations, we can speculate that the Gs-mediated effects on systolic blood pressure are indeed the result of many different mechanisms, each of which affects the complex system regulating arterial pressure in SHRs in a different way. Moreover, vascular function needs to be further investigated in the aorta and possibly in smaller renal arteries.

## 5. Conclusions

The present results show that daily oral treatment with tomato Gs significantly prevented the SBP increase, reduced behavioral hyperactivity, provided a neuroprotective action and lowered brain contents of IL-1ß and TNF-α in young SHRs. These results may be relevant to the prevention and treatment of hypertension, especially in young subjects with a significant family history of cardiovascular disease and ADHD comorbidity. Owing to its beneficial effects, a diet rich in Camone tomato could be a useful nutritional tool to support drug therapy for the restoration of normal SBP in patients.

## Figures and Tables

**Table 1:** Effects of different treatments on body weight (BW) in spontaneously hypertensive rats (SHRs) and Wistar Kyoto (WKY) rats.

Diet	WKY Weight Gain (g)	SHR Weight Gain (g)
Vehicle	41.0 ± 0.6	43.0 ± 8.0
Captopril	36.6 ± 2.3	38.0 ± 4.4
Gel/serum	39.5 ± 4.3	42.7 ± 3.6
Tomatine	39.5 ± 4.5	41.0 ± 2.9

Rats were treated with captopril, 50 mg/kg; tomatine, 8 mg/kg; Gs, 400 mg/kg. Values are mean ± SEM. No significant difference was found among the groups. ANOVA was used followed by Bonferroni post-hoc test.

## Data Availability

The data presented in this study are available on request from the corresponding author.
